# Heterogeneity in response to N‐acetylcysteine in vascular mild cognitive impairment

**DOI:** 10.1002/alz70859_107036

**Published:** 2025-12-26

**Authors:** Yejin Kang, Damien Gallagher, Nathan Herrmann, Sandra E. Black, Alex Kiss, Paul I. Oh, Joel Ramirez, Walter Swardfager, Krista L Lanctôt

**Affiliations:** ^1^ University of Toronto, Toronto, ON Canada; ^2^ Sunnybrook Research Institute, Toronto, ON Canada; ^3^ Sunnybrook Health Sciences Centre, Toronto, ON Canada; ^4^ Toronto Rehabilitation Institute, University Health Network, Toronto, ON Canada

## Abstract

**Background:**

Oxidative stress (OS) plays a significant role in vascular mild cognitive impairment (vMCI), and the antioxidant N‐acetylcysteine (NAC) was proposed to mitigate cognitive decline. However, our randomized clinical trial (NCT03306979) found no significant effects of NAC on executive function in vMCI patients. Despite this, heterogeneity in response to NAC was observed, leading to post‐hoc analyses aimed at identifying clinical predictors of NAC response.

**Method:**

A 24‐week, randomized clinical trial included participants with vMCI (<1SD population norms in executive function, memory, working memory, or processing speed with cardiovascular disease). Baseline clinical variables were selected a priori and assessed for their effects on changes in executive function z‐scores between NAC and placebo using univariate analyses. Variables with at least a 0.37 difference between their levels (cut‐off based on observed improvements in executive function across all participants: β[95%CI]=0.37[0.18‐0.56], p<0.001) were considered potential predictors of NAC response. A multivariate regression model with selected variables was used to estimate NAC’s effects under both treatments’ scenarios. An index score was calculated as the difference in predicted changes in executive function between the NAC and placebo models. Participants were grouped into quartiles according to their index scores. The treatment effect of each quartile was determined by calculating the mean difference in the empirical change in executive function between NAC and placebo groups. A 95%CI was estimated using bootstrapping with 1000 iterations.

**Result:**

A total of 53 participants (mean[SD] Age=67.7[7.9], %male=66%, BMI=27.2[5.5], MoCA=22.6[3.2]) completed the trial. Analysis found that younger age, higher BMI, non‐APOE4 carrier status, lower Apathy Evaluation Scale‐self scores, elevated homocysteine levels, and ACE inhibitor use were associated with greater improvements in executive function with NAC. Mean treatment effect [95%CI] for each quartile were: Q1=‐0.68[‐1.18,‐0.15], Q2=‐0.16[‐0.62,0.34], Q3=0.08[‐0.57,0.72], Q4=1.01[0.28,1.86]. A likelihood ratio test showed that the treatment effect significantly varied across quartiles (p<0.001). Percent responders (improvement >0.37 in executive function z‐scores) among participants who received NAC were: Q1=0%, Q2=25%, Q3=62.5%, Q4=100% (χ²(3)=10.4, p=0.02).

**Conclusion:**

These findings show significant variability in response to NAC among vMCI patients, with certain subgroups showing more pronounced benefits. This highlights the need for personalized therapeutic strategies targeting OS in vMCI.

